# Variability in Dengue Titer Estimates from Plaque Reduction Neutralization Tests Poses a Challenge to Epidemiological Studies and Vaccine Development

**DOI:** 10.1371/journal.pntd.0002952

**Published:** 2014-06-26

**Authors:** Henrik Salje, Isabel Rodríguez-Barraquer, Kaitlin Rainwater-Lovett, Ananda Nisalak, Butsaya Thaisomboonsuk, Stephen J. Thomas, Stefan Fernandez, Richard G. Jarman, In-Kyu Yoon, Derek A. T. Cummings

**Affiliations:** 1 Department of Epidemiology, Johns Hopkins Bloomberg School of Public Health, Baltimore, Maryland, United States of America; 2 Department of Virology, Armed Forces Research Institute of Medical Sciences, Bangkok, Thailand; 3 Viral Diseases Branch, Walter Reed Army Institute of Research, Silver Spring, Maryland, United States of America; 4 Division of Communicable Diseases and Immunology, Walter Reed Army Institute of Research, Silver Spring, Maryland, United States of America; University of North Carolina at Chapel Hill, United States of America

## Abstract

**Background:**

Accurate determination of neutralization antibody titers supports epidemiological studies of dengue virus transmission and vaccine trials. Neutralization titers measured using the plaque reduction neutralization test (PRNT) are believed to provide a key measure of immunity to dengue viruses, however, the assay's variability is poorly understood, making it difficult to interpret the significance of any assay reading. In addition there is limited standardization of the neutralization evaluation point or statistical model used to estimate titers across laboratories, with little understanding of the optimum approach.

**Methodology/Principal Findings:**

We used repeated assays on the same two pools of serum using five different viruses (2,319 assays) to characterize the variability in the technique under identical experimental conditions. We also assessed the performance of multiple statistical models to interpolate continuous values of neutralization titer from discrete measurements from serial dilutions. We found that the variance in plaque reductions for individual dilutions was 0.016, equivalent to a 95% confidence interval of 0.45–0.95 for an observed plaque reduction of 0.7. We identified PRNT_75_ as the optimum evaluation point with a variance of 0.025 (log_10_ scale), indicating a titer reading of 1∶500 had 95% confidence intervals of 1∶240–1∶1000 (2.70±0.31 on a log_10_ scale). The choice of statistical model was not important for the calculation of relative titers, however, cloglog regression out-performed alternatives where absolute titers are of interest. Finally, we estimated that only 0.7% of assays would falsely detect a four-fold difference in titers between acute and convalescent sera where no true difference exists.

**Conclusions:**

Estimating and reporting assay uncertainty will aid the interpretation of individual titers. Laboratories should perform a small number of repeat assays to generate their own variability estimates. These could be used to calculate confidence intervals for all reported titers and allow benchmarking of assay performance.

## Author Summary

Plaque Reduction Neutralization Tests (PRNTs) remain the most popular approach to characterize an individual's ability to neutralize dengue viruses and are widely used in both epidemiological studies and vaccine trials. However, the underlying variability in the assay is poorly understood, hindering the interpretation of PRNT titer estimates. Further, there is little standardization of its use across laboratories limiting our ability to compare results across settings. Here we used many repeated experiments on the same serum under identical laboratory conditions to estimate the variance in titer measurements. We also identified both the optimum PRNT evaluation point and statistical model to calculate titers. By providing an estimate of the variability in the assay, laboratories will be able to provide a confidence bound on individual PRNT readings. In addition by providing recommended statistical approaches that could be used across laboratories, our findings will help the standardization of the assay across settings.

## Introduction

Dengue remains a substantial public health problem in tropical and subtropical regions [1]. All four serotypes of the mosquito-borne virus are capable of producing significant morbidity and death [2]. As part of efforts to monitor and control the disease, public health agencies and vaccine developers use serological methods to perform surveillance and assess vaccine trial outcomes. A standard for characterizing serotype-specific neutralizing dengue antibody levels is the Plaque Reduction Neutralization Test (PRNT) [3]. PRNT readouts are known to vary substantially, even on samples from the same individual, however, the extent of the underlying variability in estimates remains unclear [4]. There are many potential sources of variation including within experiment and between experiment sources. In addition, different laboratories use different cell lines, different viral strains with varying viral passage number, and parametric models to calculate PRNT with the impact of the alternative approaches poorly understood [5]–[7]. Laboratories also use PRNT evaluation points that range between PRNT_50_ to PRNT_90_, and may perform varying numbers of serial dilutions [6], [8]–[10]. Understanding and characterizing the variability of the assay may greatly increase the accuracy and quantifiability of the assay, important both in epidemiological and vaccine studies.

After infection by one of the four dengue virus serotypes, individuals develop antibodies against the infecting virus [2]. The PRNT assay is used to measure neutralizing antibodies produced in response to this exposure. When an *in vitro* monolayer of cells is exposed to the virus without the presence of neutralizing antibodies, the viral particles enter and kill the cells. Where viral particles have spread between neighboring cells, a ‘plaque’ of dead cells is created that can be observed and counted. The presence of neutralizing antibodies from an individual's serum reduces the number of plaques formed by inhibiting the virus. In most cases, for a given concentration of antibodies, the addition of lower dilutions of serum result in fewer plaques formed than higher serum dilutions. PRNT_50_ is the estimated serum dilution that produces a 50% reduction in the number of plaques formed compared to the number formed on monolayers in the absence of antibody [3]. PRNT_50_ is believed to give an indication of an individual's ability to neutralize the dengue virus if exposed in vivo and to indicate whether an individual has been exposed in the past.

Absolute titer estimates from a single serum sample are used in vaccine studies as a potential marker of protection following immunization [11], [12]. In addition, epidemiological cohort studies may estimate the risk of serotype-specific disease by absolute titer at baseline [13]. Comparisons between titers from two samples taken from the same individual are also routinely undertaken. For example, four-fold differences in titers from acute and convalescent sera are typically taken to signify seroconversion [14]. Cohort studies also use large serotype-specific titer differences between study visits as evidence of infection, allowing the detection of asymptomatic infections that cannot be identified through symptomatic disease surveillance [15].

An individual's ability to successfully neutralize a strain of dengue may depend on the age of the individual, gender, nutrition, genetic factors as well as the history and time of previous infections by other flaviviruses [2], [16]. In comparing single PRNT estimates between individuals, it is not possible to separate differences due to these host factors from differences due to assay variability. Understanding the variability of the assay instead requires a large number of repeated experiments on the same serum. This necessitates large pools of serum that are rarely available. However, as part of each experiment, laboratories often use high titer and low titer serum controls to ensure consistency of experimental conditions between assays. Control sera lots can come from pooled human sera that are maintained and remain unchanged for several years. In each experiment, PRNTs are calculated for each control serum (as well as the test serum under investigation). Using the plaque counts from the control sera from a large number of assays, we can estimate the variability in the PRNT within identical experiments.

## Methods

The Armed Forces Research Institute of Medical Sciences (AFRIMS) in Bangkok, Thailand developed the dengue PRNT assay in the 1960s and has been performing it since for surveillance of dengue immunity in the population and supporting vaccine trials and cohort studies [3], [4], [13], [17]. Data for the current study comes from control assays of PRNTs performed at AFRIMS between 2007 and 2013. Briefly, in each assay, a monolayer of continuous Macaca mulatta kidney cells (LLC-MK2) was infected with virus, predetermined to be in the range of 30–50 plaque-forming units in the presence of 4-fold serial dilutions of heat-inactivated serum (range of 1∶10 to 1∶163840). The well for each dilution was 4.5 cm^2^ in size (12 wells per plate). For each dilution, the number of viral plaques was counted and compared to the number of plaques in a control where no serum was added. Each dilution and control was performed in duplicate. During the study period there were changes to the number and cell lines used to passage the virus, and the number of passages that the virus went through. In addition, the DENV-4 viral strain was changed in 2009 (Table 1). Three technicians conducted over 95% of all assays in the study period.

**Table 1:** Number of experiments by serum pool and viral strain combination.

Serum Pool		Neutralizing strain	N (%),	PRNT_75_
Serotype	High/low titer		Total = 2319	
DENV- 1	High titer	Thailand/16007/1964	288 (12)	1∶5200
DENV- 1	Low titer	Thailand/16007/1964	288 (12)	1∶510
DENV- 2	High titer	Thailand/16681/1984	279 (12)	1∶6000
DENV- 2	Low titer	Thailand/16681/1984	279 (12)	1∶650
DENV- 3	High titer	Philippines/16562/1964	285 (12)	1∶3690
DENV- 3	Low titer	Philippines/16562/1964	285 (12)	1∶320
DENV- 4A	High titer	Indonesia/1036/1976	179 (8)	1∶220
DENV- 4A	Low titer	Indonesia/1036/1976	180 (8)	1∶20
DENV- 4B	High titer	Thailand/C0036/2006	128 (5)	1∶1880
DENV- 4B	Low titer	Thailand/C0036/2006	128 (5)	1∶190

The final column shows the PRNT_75_ calculated using a smooth spline from all experiments.

### Serum pools

Two serum pools (a high titer and a low titer pool) were collected and created in 2006 and used throughout the study period. The high titer pool was obtained by pooling residual blood samples from multiple Thai individuals that tested positive for dengue virus using IgG ELISA. A portion of the pool was then diluted with human sera from PRNT-negative blood donors to create a low titer pool.

### Viruses

Five viruses were used during the study period, one each for DENV-1, DENV-2 and DENV-3 and two for DENV-4 (Table 1). Around every two years, viral stocks were generated in batches by passaging virus through C6/36 mosquito cell lines (between one and eight passages) and up to three passages in either suckling mice (SM) or LLC-MK2 cells.

### PRNT calculation

Basic regressions were used to interpolate the titer at which defined reductions (PRNT “evaluation points”) occur from the observed reductions (e.g., a 50% reduction for a PRNT evaluation point of PRNT_50_). We calculated PRNTs over the range PRNT_40_ to PRNT_90_ using either (a) probit regression, (b) logistic regression, (c) complementary log-log (cloglog) regression or (d) four-parameter non-linear regression [8]. To explore the reduction in variance from repeat dilutions, PRNTs were calculated using both individual set of dilutions and by using the average plaque reductions across repeat dilutions.

As PRNTs can be resource intensive, laboratories may perform two dilutions that they expect will contain the PRNT evaluation point of interest and use straight line interpolation on the log-transformed dilutions [6]. To estimate the variability of this approach, we initially identified the expected PRNT titer using all assays from a viral strain and serum pool and identified the two sequential dilutions that contained this value. For each experiment we then only used the values from the two sequential dilutions to calculate PRNT using straight-line interpolation. We did not calculate PRNTs in experiments where the two dilutions did not contain the PRNT evaluation point of interest.

Finally, some laboratories perform Single Dilution Neutralization Tests (SDNTs) to identify exposure using a single dilution [18], [19]. The test is scored as positive if greater than 70% plaque reduction is observed (using one or more reference dengue viruses) at a 1∶30 dilution, although the optimal plaque reduction or dilution remains unclear [18], [19]. To provide insight into the risk of incorrectly categorizing individuals as positive or negative, we calculated the variance in the neutralization proportions for all experiments from each individual dilution for each viral strain and serum pool.

### Bias and mean squared error

We assumed that the ability of each of the two serum pools to neutralize a particular viral strain was constant within any year, reflected in a single ‘true’ PRNT titer for each virus for both the high titer and the lower titer pools (i.e., one for each row in Table 1). We considered PRNT estimates from a flexible non-parametric spline, fitted to the plaque reductions from all experiments within each year from a single serum pool as the best, unbiased estimate of the ‘true’ PRNT for that pool.

We explored whether there existed any systematic differences (bias) in PRNT estimates calculated using the different models. For each experiment, we calculated PRNT titers using each of the models (probit, logit, cloglog regression and non-linear regression). Bias was suggested when there was a systematic difference between the PRNT estimates using the model and the estimate of the ‘true’ titer. In addition we calculated the mean squared error (MSE) in the estimates. We reported an average MSE, bias and variance for each PRNT evaluation point and model, weighted by the number of experiments using each virus and serum pool. We used bootstrapping to generate 95% confidence intervals for the bias, variance and MSE estimates from each PRNT evaluation point and model. Over 1,000 resamples, we recalculated the bias, variance and MSE of the assays. Ninety-five per cent confidence intervals were calculated from the 2.5% and 97.5% quantiles from the resultant distributions. Finally, to explore whether time difference between assays was associated with observed variability, we estimated the variance in titers for assays performed across different time periods.

### Plate-specific versus non-plate-specific sources of variance

We can divide variability in titers into ‘plate-specific’ and ‘non-plate- specific’ sources of variance, where ‘plate-specific’ is taken to mean experimental factors that are identical in assays performed on the same plate. In particular this will include the preparation of diluted virus solution that is added to wells on the same plate (a new viral solution is made for each separate plate). Non-plate-specific factors are all other sources of variability that will be present across all assays, irrespective of whether they are conducted on the same plate or not. This will include variability in the ability of the serum to neutralize the virus and variability in plaque counting. We estimated non-plate-specific variability by calculating the variance in titer estimates across assays performed on the same plate. Plate-specific variance was calculated by subtracting the non-plate-specific variance from the total variance in titers from assays performed on difference plates.

### Absolute and relative titer measurements

Absolute antibody titers may be used as a marker of immunity. More common, however, is the comparison of titers from convalescent and acute sera taken from the same individual for detection of seroconversion. Increasing the number of repeat dilutions for each serum will reduce the uncertainty of both absolute and relative titer estimates. Using our estimates of the variability in the assay, we calculated the expected variance from performing different numbers of repeat dilutions (varied between 0 and 3 repeats). We considered scenarios where repeated dilutions were conducted on the same plate (and would therefore not reduce plate-specific sources of variability) and where repeats were conducted on different plates where both plate-specific and non-plate-specific variance would decrease. In addition we used our variance estimates to calculate the proportion of paired samples that would result in a greater than four-fold difference in titers where no true difference exists (i.e., a false positive result).

### Multilevel model

Plaque density may be associated with differential levels of plaque overlap, which would bias titer estimates. We can use the number of plaques in the reference well (where no sera is added) as a marker of plaque density. Heterogeneities in the passaging of the virus may also be associated with changing PRNT estimates. To quantify systematic differences in titer estimates by the number of passages and the type of cell (C6/36, SM and LLC-MK2 cells), plaque density and the age of the virus stock, we constructed a multilevel model with a random intercept for each viral strain and serum pool combination (listed in Table 1).

### Ethics statement

All experiments were conducted using pooled residual sera from public health service testing and, as per Walter Read Armed Institute of Research (WRAIR) policy, did not require ethics review. WRAIR is the parent organization of AFRIMS.

Detailed methods can be found in the supplementary materials.

## Results

Between 2007 and 2013, a total of 2,319 assays were performed using five different viruses on two different control sera (Table 1). An average of 4.4 dilution steps were performed in each assay (range: 3–5) with each dilution performed twice (20,286 individual dilutions in all).

### Plaque reduction variability

**Figure 1:**
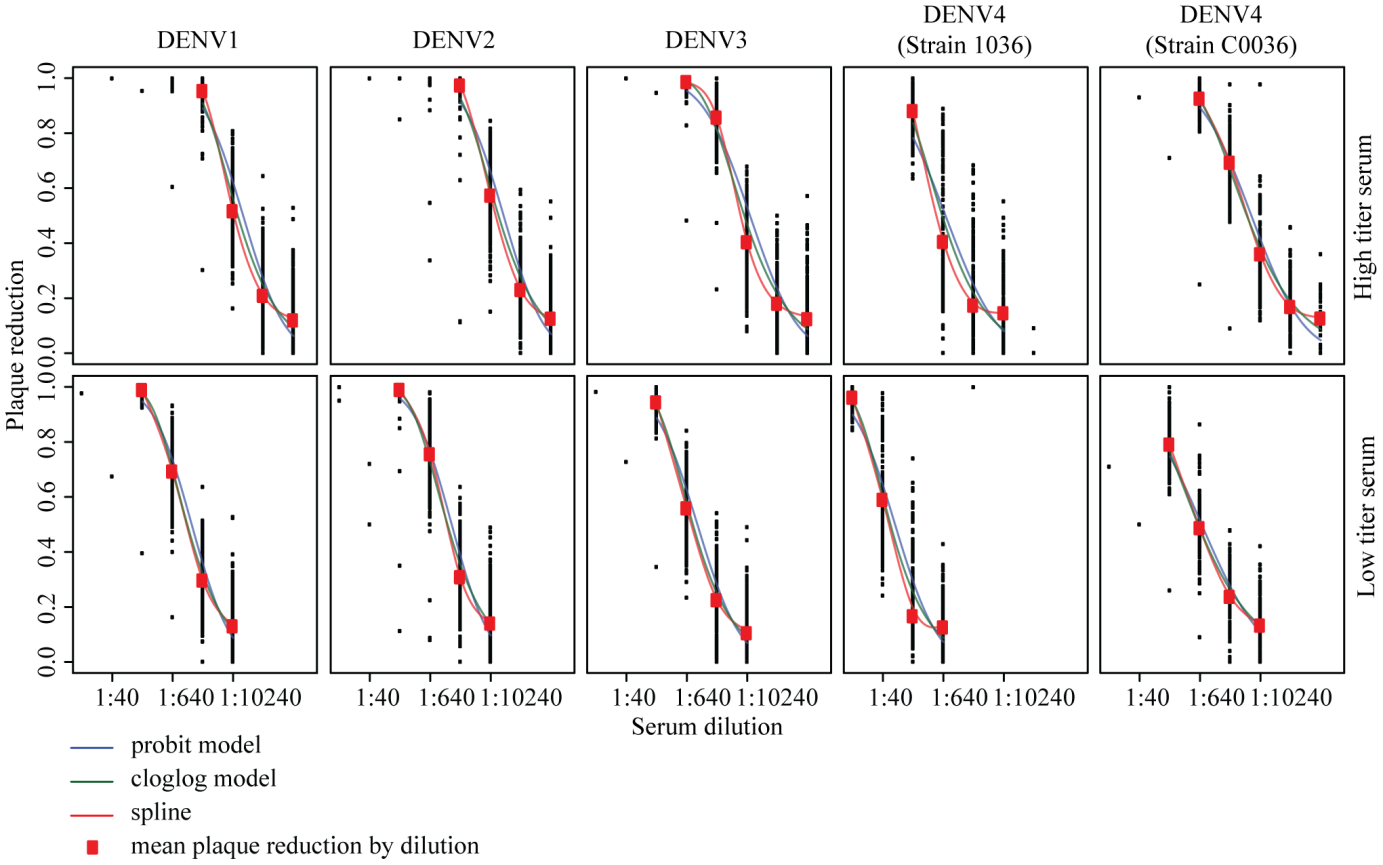
Plaque reduction estimates for each experiment. Each black dot represents the mean reduction in plaques formed for that dilution from two repeats. The red dots are the overall means across all the experiments. Superimposed are fitted models using a probit transformation, a cloglog transformation and a non-parametric spline (the estimate of the ‘true’ titers).

**Figure 2:**
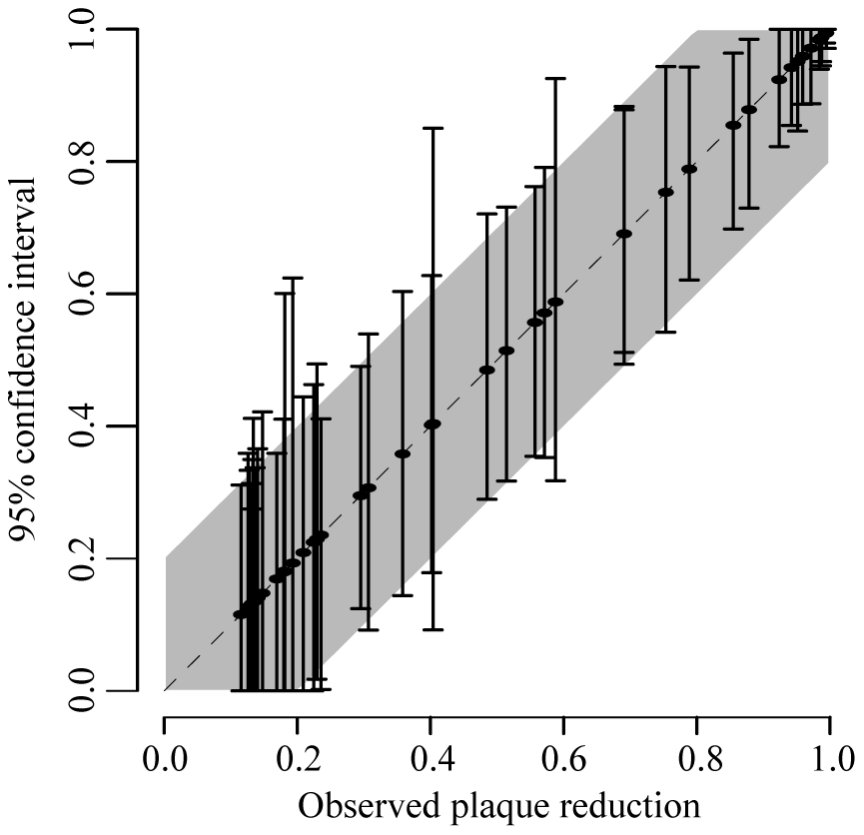
Plaque reduction proportions for individual dilutions. Each solid line represents the 2.5% and 97.5% quantiles of plaque reductions from the average of two repeats from a single dilution from a particular virus – serum pool combination. The shaded area represent asymptotic 95% confidence intervals calculated from the overall variance observed in the plaque reduction proportions.

There existed substantial variability in the plaque reduction proportions (Figure 1) with consistent variability in plaque reductions for each dilution (Figure 2). On average, the variance for an individual dilution performed on the same serum using the same virus was 0.016, equivalent to a 95% confidence interval of 0.45–0.95 for an observed plaque reduction of 0.7. Performing a repeated set of dilutions reduced the variance to 0.011, equivalent to a 95% confidence interval of 0.50–0.90 for the same observed plaque reduction of 0.7.

### Identifying the optimum PRNT model and PRNT evaluation point

**Figure 3:**
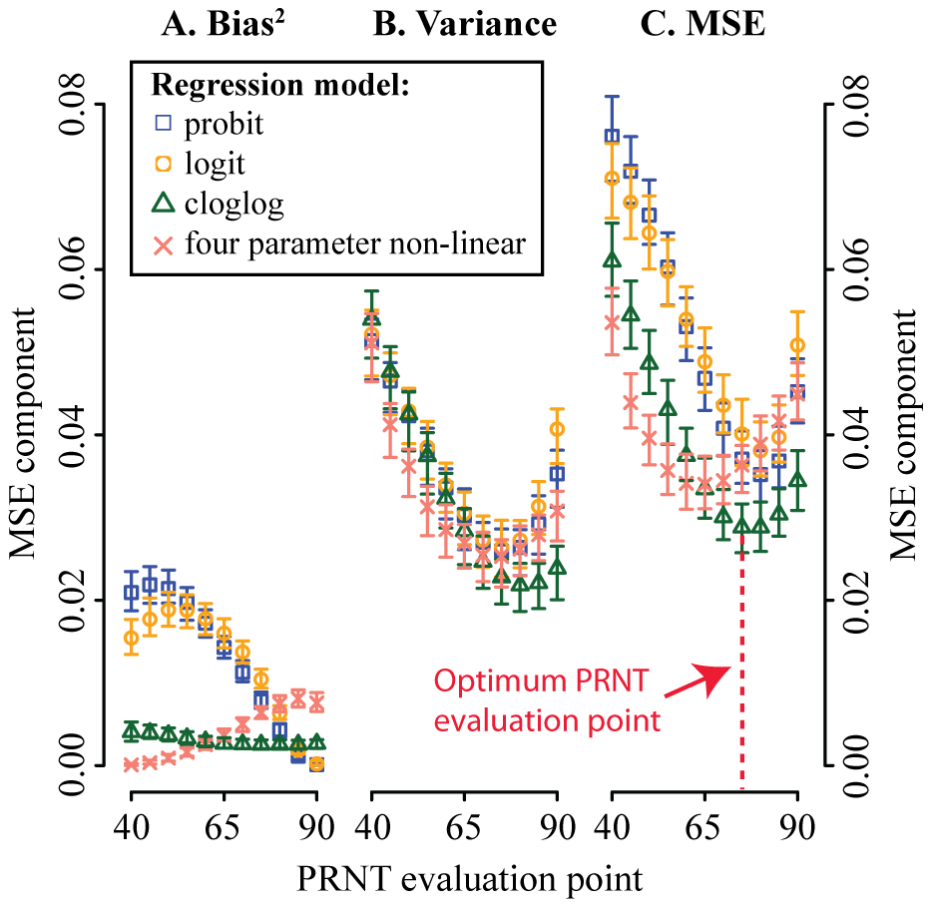
Estimates of (A) bias^2^, (B) variance and (C) mean squared error in titers from a single set of dilutions by PRNT evaluation point for the different models. Bias for each experiment was calculated by comparing the model PRNT results with that from a smooth spline from all experiments from that particular virus and serum pool. Only assays where titers could be calculated for all four models (63% of all assays) were used.

The variability in plaque reduction proportions led to heterogeneity in PRNT titer estimates. For each set of dilutions performed on each serum and using each virus, we used four different statistical models to calculate titers at PRNT evaluation points varying from PRNT_40_ to PRNT_90_. In each case we calculated the bias, variance and mean squared error in the estimated titers. Four-parameter non-linear regression could only be used on 63% of the assays, as the remaining assays had either insufficient dilutions or the resultant curve fluctuated too much to allow model fit (Table 2). All assays could be used for the other statistical models. When comparing the models, we only used the assays where estimates existed for all four approaches. We found that the probit and logit models consistently over-estimated titers (Figure 3A). For example, for a PRNT evaluation point of PRNT_50_, the probit model overestimated the titer by an average of 0.14 (log_10_ scale) and logit by 0.12 (log_10_ scale). The cloglog and four-parameter non-linear regression were largely unbiased. The variance in titer estimates was very similar across models (Figure 3B). However, the variance varied widely by PRNT evaluation point. Variance was lowest at PRNT_75_ for the probit, logit and four-parameter non-linear regression and lowest at PRNT_80_ for the cloglog model. Overall, the lowest mean-squared error existed from using the cloglog model at a PRNT evaluation point of PRNT_75_ (Figure 3C).

**Table 2:** Estimated standard deviation and bias in PRNT_75_ estimates using the different models.

Model	Proportion of assays	Variance (log_10_ scale)	Bias (log_10_ scale)
		[95% confidence interval]	
Probit regression	100%	0.036 [0.030–0.040]	0.042 [0.035–0.049]
Logistic regression	100%	0.036 [0.030–0.040]	0.055 [0.049–0.063]
**Cloglog regression**	**100%**	**0.025 [0.021–0.030]**	**0.027 [0.021–0.033]**
*Four-parameter non-linear regression*	*63%*	*0.022 [0.017–0.027]*	*0.059 [0.052–0.066]*

Values are averages from the different viruses and serum pools, weighted by the number of experiments.

Where only two dilutions were used, only 50% of the experiments could be used as the two sequential dilutions did not contain the PRNT evaluation point in the remainder and would have required extrapolation. Where it could be estimated, the standard deviation of PRNT_50_ using two dilutions was estimated at 0.13 (log_10_ scale), however, this only represents the variability of the subset of the experiments where the two dilutions had reductions in plaques that were closest to the best estimate of the unbiased PRNT and cannot be compared to the variability observed in the other approaches.

### Plate-specific versus non-plate-specific sources of variance

To calculate the variance in non-plate-specific factors, we calculated the variance in titers calculated from single sets of dilutions performed on the same plate using a cloglog model and a PRNT evaluation point of PRNT_75_ (the model and evaluation point with the lowest mean squared error) from all assays. We found a variance of 0.014 (log_10_ scale) for titer estimates calculated on the same plate compared to a variance of 0.032 (log_10_ scale) for titer estimates from different plates (all estimated from single sets of dilutions). These findings indicate that the variance from non-plate-specific factors was 0.014 (log_10_ scale) and the variance from plate-specific factors was 0.018 (the difference in the two variance estimates, log_10_ scale). We found no difference in the variance in titers between assays by the time separation between them: titers from assays performed within the same month were as variable as from assays performed over a year apart (Figure S1). When a single titer estimate was calculated from two sets of dilutions from the same plate (i.e., the common practice of performing a repeat set of dilutions on the same plate), the variance in titer estimates was 0.025 (log_10_ scale). This is identical to what we would expect from a reduction in non-plate-specific variance only (i.e., 0.018+0.014/2). Had repeats been performed on different plates, we estimate that the variance would have reduced further to 0.016 (i.e., 0.018/2+0.014/2, log_10_ scale) reflecting a reduction in both non-plate-specific and plate-specific variance.

Laboratories may wish to calculate their own lab-specific variability measures by performing repeated assays on the same serum. To estimate the number of assays laboratories would need to perform to get a reliable variance estimate, we estimated the precision in the variance estimate using different numbers of assays (between 2 and 30) on the same serum using the same viral strain. We found that with only 20 assays, the width of the 95% confidence interval for the variance would be only 0.013, only slightly higher than the width calculated from all samples (Figure S2).

### Absolute titer measurement

**Figure 4:**
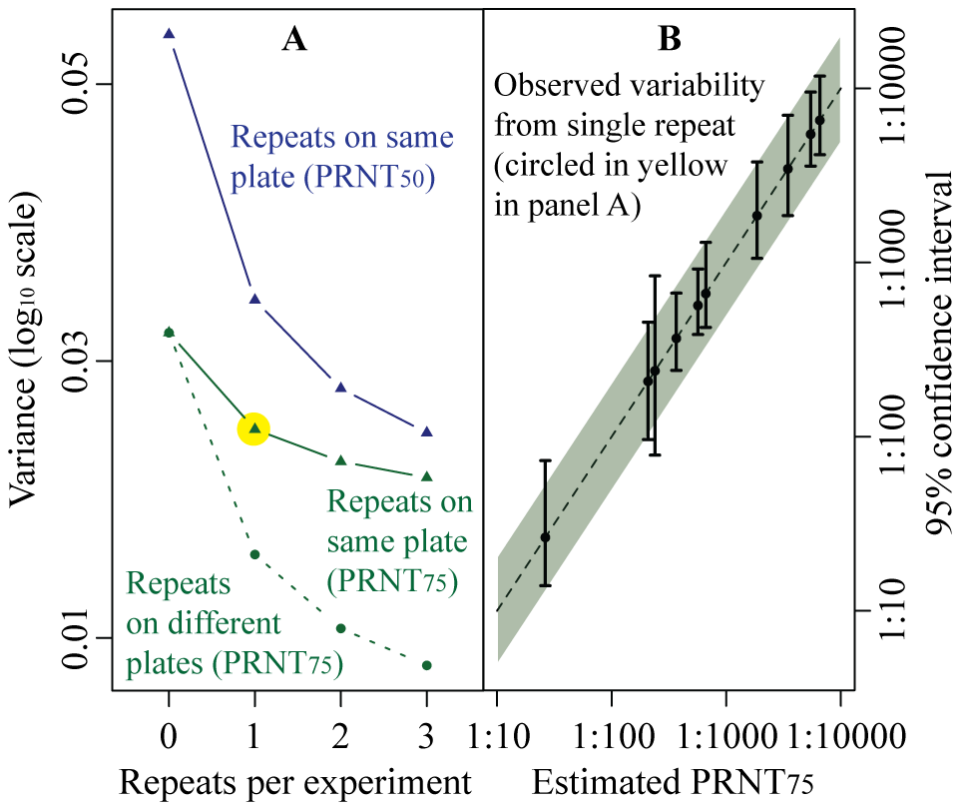
(A) Variance in absolute titers by the number of repeat sets of dilutions and where the repeats are performed (either the same plate or on different plates). All titers estimated using cloglog regression. (**B**) Observed titer variability. Each solid line represents the 2.5% and 97.5% quantiles from the titers estimated from the serum pool – virus combinations listed in Table 1. The shaded area represents asymptotic 95% confidence intervals calculated using a single variance estimate from all assays (represented by the yellow circle in panel A).

We found that the titers estimated for each of the ten virus- serum pool combinations varied similarly (Figure 4B). Therefore a single variance estimate appears appropriate when calculating confidence intervals for a titer. We found that, for example, for a titer estimate of 1∶500 where no repeat dilutions have been performed and the titer was estimated using a cloglog model with a PRNT evaluation point of PRNT_75_, a 95% confidence interval would be 1∶200–1∶1100 (2.70±0.35 on a log_10_ scale). Performing a repeated set of dilutions on the same plate (the most common practice) results in a confidence interval of 1∶240–1∶1000 (2.70±0.31 on a log_10_ scale). Finally, performing repeated dilutions on a separate plate reduces the confidence intervals further to 1∶280–1∶880 (2.70±0.25 on a log_10_ scale). Note that performing a single repeat on a different plate reduces the width of the confidence intervals substantially greater than performing even additional repeats on the same plate (Figure 4A). We tested the coverage of our uncertainty estimates by calculating individual confidence intervals for each assay (2,319 confidence intervals in all). We found that 96% of these intervals contained the true titer estimates, suggesting excellent coverage.

### Relative titer measurements

**Figure 5:**
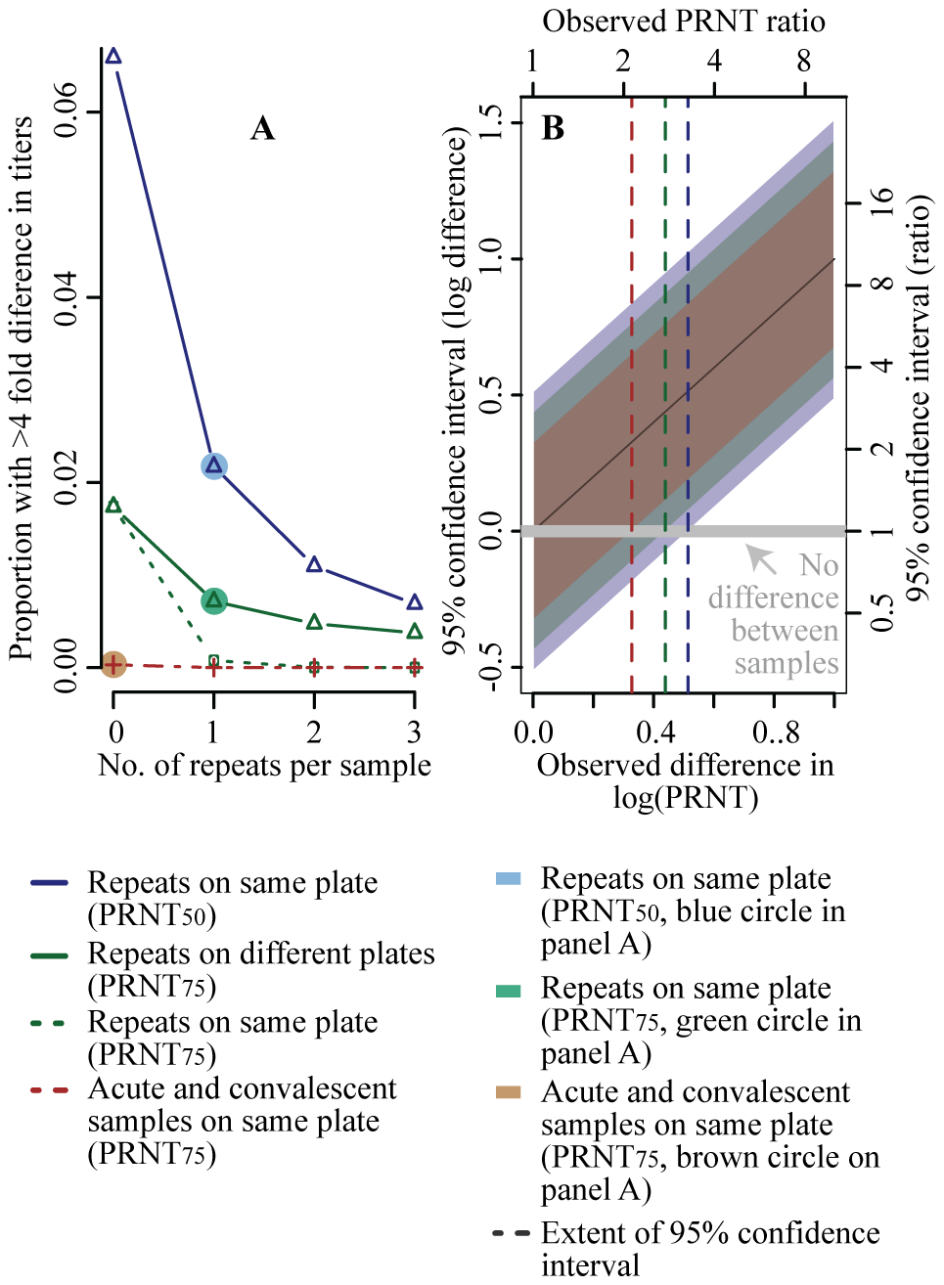
(A) Probability of detecting a greater than four-fold difference in titers between paired sera where no true difference exists. All titers calculated through cloglog regression. (**B**) Example confidence intervals for the log difference between titers calculated from paired sera (and the equivalent intervals for the ratio) under different scenarios of PRNT evaluation points and where repeat dilutions are performed.

Researchers are often interested in the ratio of titers between acute and convalescent serum samples taken from the same sick individual. We found that while the probit and logit models produced substantially biased results, the extent of the bias did not appear to differ by the magnitude of the titer (Figure S3). Therefore, while individual PRNT estimates may be biased using these models, ratios of PRNTs would not be as both the numerator and the denominator would be similarly biased. As the different statistical models had similar levels of variance (Figure 3B), the choice of model when calculating relative titers is less important. The PRNT evaluation point, however, is crucial to minimizing variability, with a PRNT_75_ evaluation point up to 50% less variable than alternatives (Figure 3B). Four-fold differences or greater in titers are often used as evidence of seroconversion. Using our variability estimates, we calculated the probability of detecting a greater than four-fold difference in titers where there was no difference in true titers (i.e., a false positive result). We found that when a single set of dilutions is performed and a PRNT evaluation point of PRNT_75_ is used, only 1.7% of assays would falsely detect a greater than four-fold difference in titers compared to 6.4% with a PRNT evaluation point of PRNT_50_ (Figure 5A). Performing a single set of repeat dilutions on the same plate (but with the acute and convalescent samples still performed on separate plates) reduced the probability further, to 0.7%. Finally, if both the acute and convalescent samples are on the same plate, the probability of falsely detecting a greater than four-fold difference in titer was 0.03%. Figure 5B sets out the 95% confidence intervals for different observed ratios: for example the 95% ratio for an observed two-fold difference in titers using a PRNT evaluation point of PRNT_75_ is 0.7–5.5 (equivalent to 0.3±0.4 on a log_10_ scale).

### Multilevel model

To estimate the effects of experimental conditions on titers we built a multilevel model incorporating the number of viral passages, the cell type, reference well plaque count and the age of the virus stock used in the experiments. We found that passaging the virus in SM increased titers compared to LLC-MK2 cells (effect size of 1.15, 95% confidence interval of 1.09–1.21). The total number of passages and the age of the viral stock at the time of the experiment did not affect the titers. Higher plaque counts in the reference well was associated with a very small reduction in titers (effect size of 0.99, 95% confidence interval of 0.98–99). Less than 0.7% of the variability in PRNT_50_ estimates could be explained by the model covariates, leaving over 99% of variability unexplained (Table 3).

**Table 3:** Results of multilevel model for impact of experimental factors on PRNT_75_ estimates using cloglog regression.

Parameter		Coefficient [95% CI]
Age of virus stock (yrs):	mean: 2.2, sd: 1.0	1.00 [0.98–1.01]
# Plaques in reference well:	mean: 32.6, sd: 7.1	0.99 [0.98–0.99]
Total # of passages:	mean: 5.3, sd: 2.1	0.99 [0.98–1.00]
Cell passage:		
- C6/36 and LLC-MK2	# experiments: 502	Ref
- C6/36 and SM	# experiments: 1566	1.15 [1.09–1.21]
- Only C6/36	# experiments: 262	0.92 [0.06–15.2]
R^2^ (1)		0.006

(1) Marginal R^2^ that indicates the proportion of variance explained by the fixed effects only [31]. Model also adjusted for year of assay.

The model has a random intercept for the viral strain and serum pool used in the experiment and is also adjusted for the year of the assay. All coefficients have been transformed by raising 10 to the power of the coefficient.

## Discussion

Using repeated assays on the same serum sample with the same viral strain, we estimated the extent to which measured PRNTs vary. We found a consistent level of variability in titer estimates across the viruses and serum pools used during the study period. A measure of the variability of titers provides information on the potential misclassification of individuals falling above or below any specified PRNT evaluation point, information routinely used in calculating sample sizes for a wide range of studies. By characterizing the variability in measured titers, these findings will aid in the determination of an individual's immunity, the design and interpretation of results from immunogenicity trials, epidemiologic studies and allow the benchmarking of assays across laboratories. Based on our findings, we set out a number of recommendations for laboratories performing PRNTs (Box 1).

**Box 1. Recommendations for Performing and Calculating Absolute and Relative PRNT Titers**

**Laboratory benchmarking**

1. Perform repeated assays (∼20) on same serum to quantify lab-specific assay variability(a)

**Absolute titers**

1. Where possible, perform repeat dilutions on separate plates with separate viral preparations
2. Calculate titers using cloglog regression at a PRNT evaluation point of PRNT_75_
3. Report log-transformed titers with uncertainty estimates calculated from benchmarking exercise
4. Report raw count data with dilutions in supplementary materials

**Relative titers**

1. Where possible, place paired samples on same plate
2. Calculate individual titers at a PRNT evaluation point of PRNT_75_ (choice of model is less important)
3. Report difference of log-transformed titers with uncertainty estimates calculated from benchmarking exercise
4. Report raw count data with dilutions in supplementary materials

(a) Where laboratories use identical sera as controls in each assay, the variability in those titers could be used instead.

There have been a number of efforts to standardize the assay [7], [20]–[24]. A comprehensive effort by Roehrig et al., set out guidelines for PRNTs, which were supported by the World Health Organization [20], [21]. Nevertheless, heterogeneities in approaches between laboratories persist, particularly in PRNT evaluation points, in large part because there had been no comprehensive study of the variability in titers. The WHO recommends using a PRNT_50_ titer for vaccinee sera and PRNT_90_ titers for epidemiological studies. However, both vaccine and epidemiological studies regularly use alternative PRNT evaluation points [6], [10]. The stated benefit of the higher evaluation point is to decrease variance while the stated benefit of the lower evaluation point is of increased accuracy (i.e. reduced bias) [21]. We found that the evaluation point with the lowest variance was actually between PRNT_75_ and PRNT_80_ with little difference between the different models used to calculate the titers. Bias, by contrast, differed substantially by model with four-parameter non-linear regression and cloglog largely unbiased across PRNT evaluation points whereas probit and logit regression consistently over-estimated titers. However, the choice of model only appeared important where absolute titers were of interest as biases cancelled out in the calculation of relative measures. Overall, we found that a PRNT evaluation point of PRNT_75_ minimized the MSE between the model PRNT estimates and our best estimate of the unbiased PRNTs and should be used, where possible, across study types. Lower PRNT evaluation points may remain preferable in the estimation of low titers where the estimate at PRNT_75_ can be regularly below the limit of detection (typically 1∶10). However, the biological meaningfulness of low PRNT titers is currently under scrutiny. A recent dengue vaccine trial observed infections in vaccinated individuals despite the apparent presence of detectable titers [25]. These findings suggest that ‘some’ titers (e.g., greater than 1∶10) versus ‘no’ titers (e.g., less than 1∶10) is insufficient to differentiate between individuals with and without protective immunity. Further research is urgently required to understand if vaccines need to elicit higher titers than those generated by the trial vaccine or if there exist qualitatively different markers of protection (such as T-cell responses).

We found that around half of the variance in titers could be explained through plate-specific factors, experimental conditions that differ between plates but not within plates. In particular, creating the viral preparations for each plate may be an important contributor. Performing repeat dilutions on the same plate cannot reduce plate-specific variance, as both repeats will be perfectly correlated for these factors. This explains why there was only a small reduction in variance in titers calculated from repeat dilutions compared to the variance from only single sets of dilutions (0.025 versus 0.032). Performing repeats on separate plates would reduce this further (we estimate to 0.016) and should be considered where precise absolute titers are required. Where relative titers are calculated, we estimated that the probability of observing four-fold differences in titers where it does not truly exist is less than one per cent when a PRNT evaluation point of PRNT_75_ is used (it is twice as high when a PRNT_50_ evaluation point is used). Our findings indicate that the risk of a false positive detection of a significant difference in titers is low even where a cut-off of a three-fold difference in titers is used, especially where both the acute and convalescent sera are placed on the same plate. It remains unclear whether such titer differences are correlated with immune protection or past exposure.

Laboratories may perform only two dilutions and use linear interpolation to obtain PRNT estimates. We found that we could only use half of the assays for this analysis, as the remaining experiments would require unwise extrapolation outside the results from the two dilutions. In these situations, laboratories need to repeat the assays at wider dilution ranges. The substantial number of experiments that could not be included in the analysis suggests that performing only two dilutions may only have minimal benefits. Single dilution neutralization tests only require a single dilution, however, individual plaque reduction estimates had wide confidence intervals: individuals with a true plaque reduction of 75% (and therefore should be scored as ‘positive’ in a SDNT using an evaluation point of 70%) had 95% confidence intervals of 54%–96%, suggesting that many such individuals would be wrongly characterized as negative [18], [19]. This provides no insight into the sensitivity or specificity of detecting exposure from using an evaluation point of 70%.

The DENV-4 strain used in the assays was changed in 2009 resulting in a 11.4-fold increase in mean titers in the high serum pool and a 12.1-fold increase in the low serum pool, confirming previous findings of the importance of viral strain on titer estimates [4]. The two DENV-4 strains come from two different genotypes (the earlier strain was genotype 2 whereas the later one was genotype 1). These findings highlight the possibility of vastly different immunological response even within a single serotype. Alongside the effect of viral strain, it has been suggested that the number and cell type of viral passages could produce systematic differences in PRNT estimates [4], [7]. We found a small increase in titers in experiments using viruses passaged through SM compared to LLC-MK2 cells supporting similar previous findings [4]. The total number of viral passages did not appear to impact PRNT estimates, however, only small numbers of passages were conducted (maximum of eight). Increasing this substantially or only using mammalian cell lines such as Vero cells as recommended by the WHO, may nevertheless impact estimates. The presence of many overlapping plaques in a well may lead to under-estimates in true plaque counts. However, we found only negligible difference in titer estimates by the number of plaques in the reference well, suggesting plaque overlap did not affect our results. These findings suggest that the under-estimate in true plaque counts was consistent across dilutions; alternatively, the wells were sufficiently large and the plaque counts sufficiently small to avoid substantial overlap. Laboratories using smaller wells may nevertheless experience titer differences from differential levels of plaque overlap by dilution. Overall, aside from viral strain, experimental factors varied in our assays explained less than one per cent of the observed variability in titer estimates. Experimental factors that were held constant throughout our experiments, such as incubation time and plaquing cell line, may nevertheless impact titer estimates.

Our findings show that the assay is inherently variable. There are many potential sources of variability in each experiment: (a) the number of viral particles pipetted into each plate, (b) the extent of viral–antibody interaction (c) the spatial arrangement of cells in the monolayer and (d) the number of non-overlapping plaques successfully generated and counted. While technicians can minimize differences through effective mixing and careful dilutions, there may be a limit to the extent that variability in these factors can be reduced. The use of automated counting methods that allow faster and more accurate particle counting may help [26]. A related approach, the flow reduction neutralization test that relies on immunofocus rather than cell death, may produce less variable titer estimates [27]. In addition, flow-based methods in laboratories with access to flow cytometry equipment show some encouraging results, especially as these methods can use human cells and allow for high-throughput of samples [26], [28]–[30]. Further work is needed to quantify the variability of these alternative approaches.

The serum pools come from pooled human sera that contain a wide range of antibodies not representative of a single individual's serum. Nevertheless, the ability for the pooled serum to neutralize a single virus should remain constant. We were not able to explore the biological significance of individual titers. In particular, the significance of low titers for immune status remains unclear, as does the serotype-specificity of the assay. PRNTs are used to characterize infection parity, with high titers against two or more serotypes considered suggestive of secondary infection. Future research using sera of known infection status could shed light on the specificity of such classifications. Further, serum with neutralization titers outside the range used in this study may perform differently. The range of titers in this study was wide (PRNT_75_ range of 1∶20–1∶6000) and we observed a consistent pattern in variability across this range. Nevertheless, naturally occurring low titer antibodies (rather than the diluted high titer pools used here) may have different levels of avidity and affinity that could impact titer variability. All viruses were passages through C6/36 cells. Viruses solely passaged through mammalian cells may be differently neutralized. We only used LLC-MK2 monolayers as plaquing cells. Other laboratories use different cell lines (such as Vero cells, recommended by the WHO or BHK cells), which may behave differently. However, it is unlikely that the variability in titers would be markedly different. Laboratories that use markedly different protocols may identify different optimal PRNT evaluation points. These could be identified using the methods presented here on repeated assays on the same sera.

In conclusion, providing uncertainty estimates with both absolute and relative titer estimates would greatly aid the interpretation of individual read-outs. While the estimates provided here provide a first marker of the variance in the assay, heterogeneities in variability between laboratories will exist. By performing a small number of repeat assays (20 appears to be sufficient to obtain a precise variance estimate) on the same serum with the same virus on different plates, laboratories could generate lab-specific variability estimates without requiring excessive resources. Alternatively, where assays on identical control serum are performed as routine, the variance in titers from these assays could be calculated instead. Variance estimates could then be used to calculate confidence intervals for all reported titers and allow benchmarking of assay performance. This study demonstrates the utility of raw results. Laboratories should consider reporting plaque counts alongside titer estimates. This will allow investigators to easily compute alternative titers using different PRNT evaluation points or statistical models, facilitating comparison across laboratories. We also recommend that titers be reported on a logarithmic scale (or log differences for relative titers) to allow easy calculation and interpretation of confidence intervals.

## Supporting Information

Data S1 Count data from all experiments.
(CSV)

Figure S1 Variance in titers between assays over different time lags between the assays. All titers calculated using a PRNT evaluation point of PRNT_75_ using cloglog regression from a single set of dilutions.
(TIF)

Figure S2 Width of variance 95% confidence interval with different numbers of repeated assays. Over 1,000 simulations between 2 and 30 titers were randomly sampled from all the titers (using cloglog regression at a PRNT evaluation point of PRNT_75_) calculated from a randomly chosen viral strain – serum pool combination from a single year. The variance between the titers was then calculated. The line represents the width of the 95% confidence interval calculated from the 2.5% and 97.5% quantiles from the resultant distribution.
(TIF)

Figure S3 Variability in the bias in (**A**) PRNT_50_ using conventional probit regression and (**B**) PRNT_75_ using cloglog regression by titer (log_10_ scale). The red dots represent the mean bias from each serum pool.
(TIF)

Text S1 Detailed methods.
(DOCX)
