# Supplementary figures and images for: Variability in Dengue Titer Estimates from Plaque Reduction Neutralization Tests Poses a Challenge to Epidemiological Studies and Vaccine Development
Source: PLoS Negl Trop Dis. 2014 Jun 26;8(6):e2952. doi: 10.1371/journal.pntd.0002952 (PMC4072537; doi:10.1371/journal.pntd.0002952)

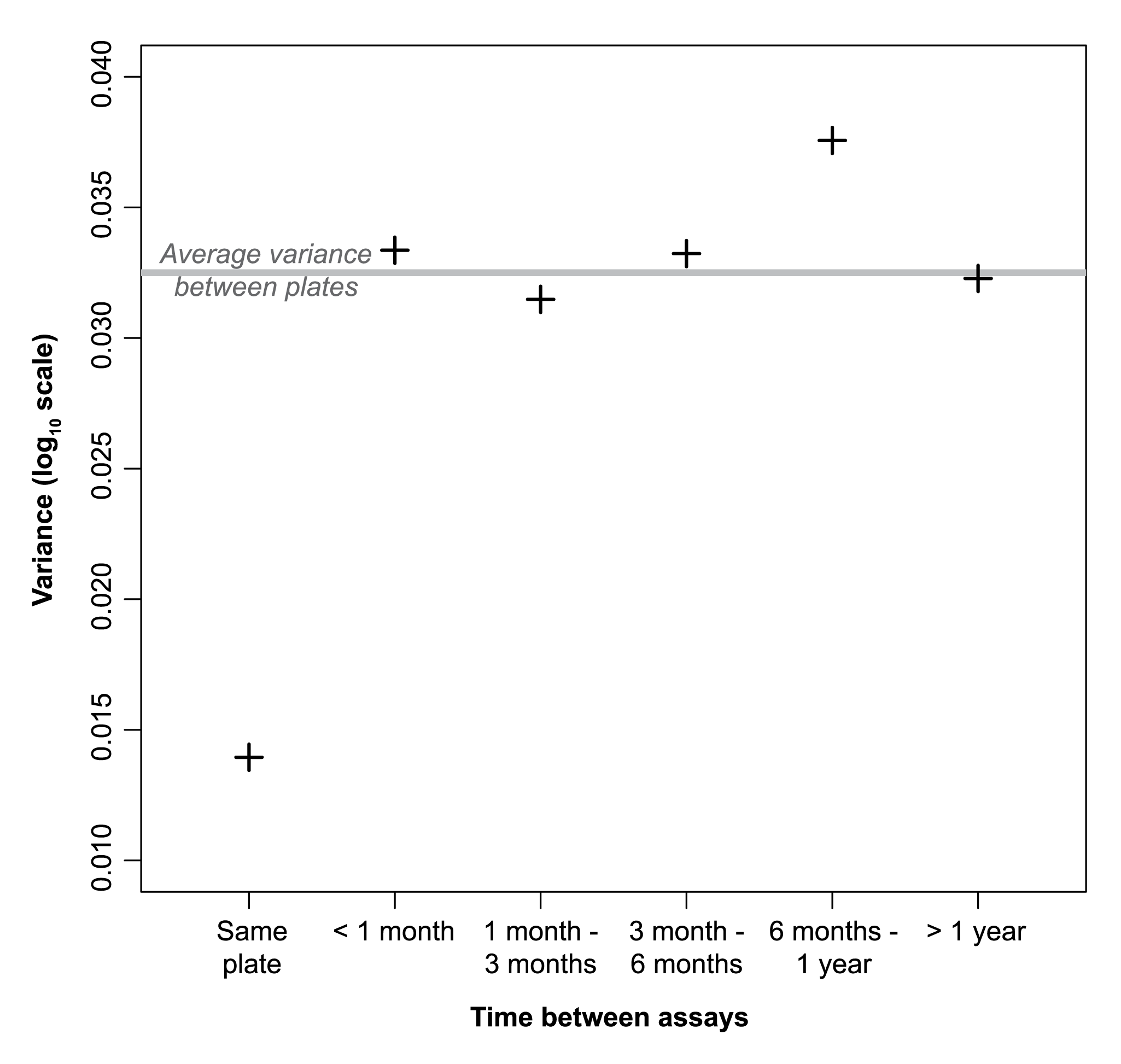

Supplement: Figure S1 — Variance in titers between assays over different time lags between the assays. All titers calculated using a PRNT evaluation point of PRNT75 using cloglog regression from a single set of dilutions. (TIF) [file pntd.0002952.s002.tif]

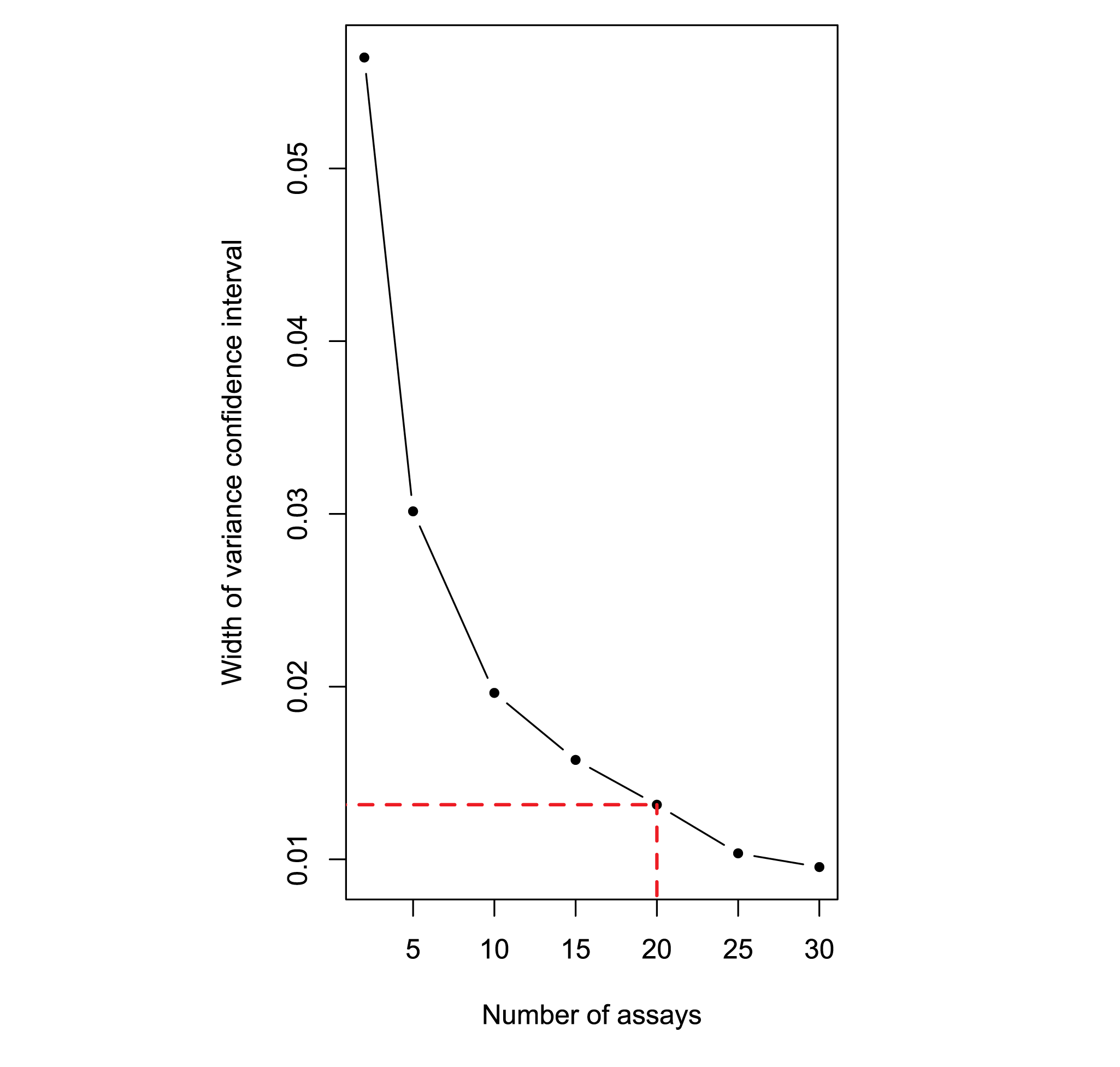

Supplement: Figure S2 — Width of variance 95% confidence interval with different numbers of repeated assays. Over 1,000 simulations between 2 and 30 titers were randomly sampled from all the titers (using cloglog regression at a PRNT evaluation point of PRNT75) calculated from a randomly chosen viral strain – serum pool combination from a single year. The variance between the titers was then calculated. The line represents the width of the 95% confidence interval calculated from the 2.5% and 97.5% quantiles from the resultant distribution. (TIF) [file pntd.0002952.s003.tif]

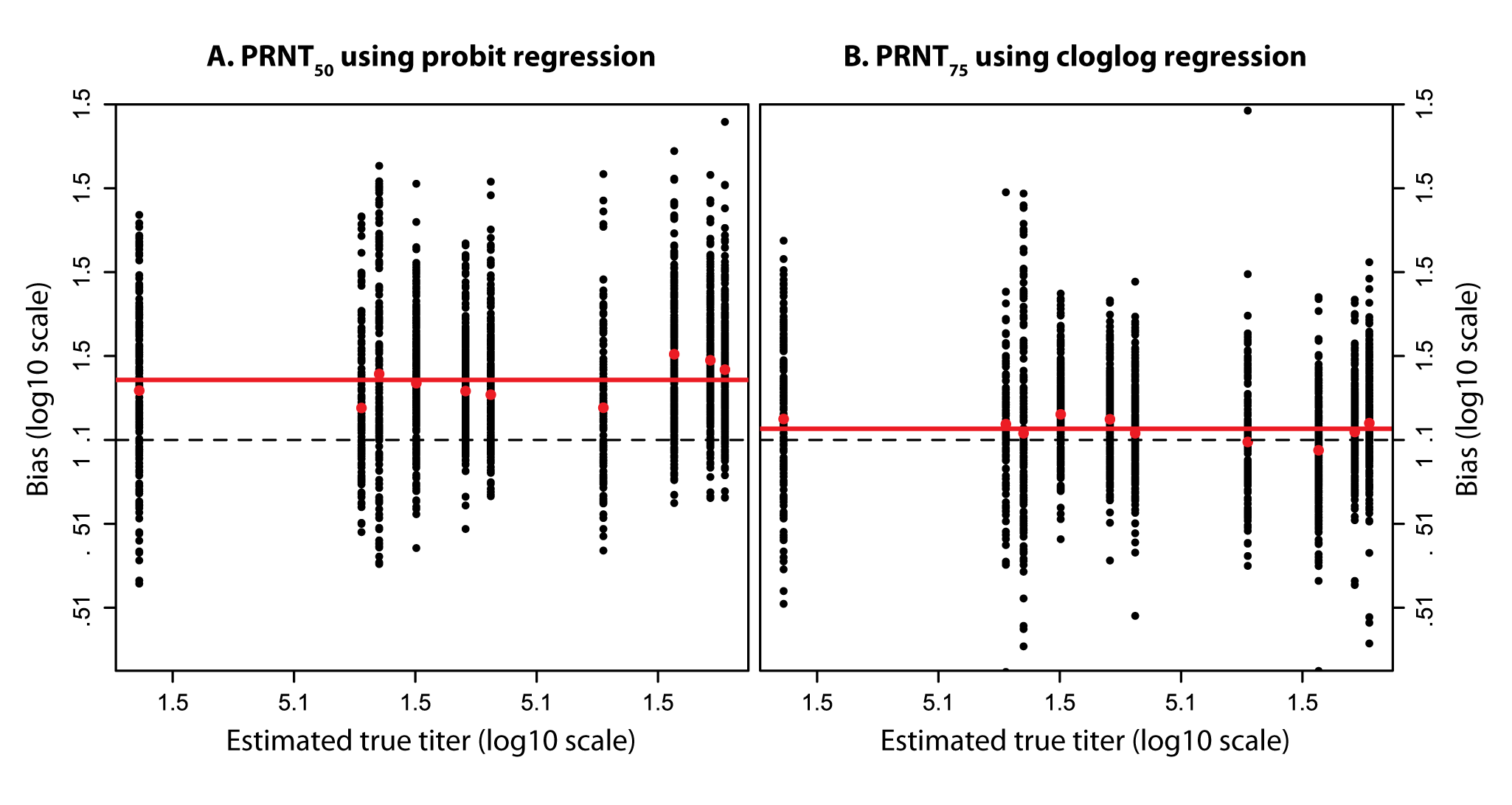

Supplement: Figure S3 — Variability in the bias in (A) PRNT50 using conventional probit regression and (B) PRNT75 using cloglog regression by titer (log10 scale). The red dots represent the mean bias from each serum pool. (TIF) [file pntd.0002952.s004.tif]
