# Supplementary material for: Variability in Dengue Titer Estimates from Plaque Reduction Neutralization Tests Poses a Challenge to Epidemiological Studies and Vaccine Development
Source: PLoS Negl Trop Dis. 2014 Jun 26;8(6):e2952. doi: 10.1371/journal.pntd.0002952 (PMC4072537; doi:10.1371/journal.pntd.0002952)
Supplement: Text S1 — Detailed methods. (DOCX) [file pntd.0002952.s005.docx]

**Text S1. Detailed methods**

***PRNT calculation***

The plaque reduction proportion for each dilution (*p_d_*) was calculated as:

$$p_{d}=1-\frac{n_{d}}{n_{0}}$$

where *n_d_* number of plaques at dilution *d* and *n_0_* is the number of plaques formed when no sera is added. Titers can be estimated using generalized linear regression using the log-transformed dilutions and either a probit, logit or complementary log-log (cloglog) link function:

1. probit:

$$probit\left( p_{d} \right)=\phi^{-1}(p_{d})$$

2. logit:

$$logit\left( p_{d} \right)=\log\left( \frac{p_{d}}{1-p_{d}} \right)$$

3. cloglog:

$$cloglog\left( p_{d} \right)=log(-log(1-p_{d}))$$

where $\phi^{-1}$ is the inverse cumulative distribution function of the standard normal distribution. These regressions are used to interpolate the titer at which defined reductions occur from the observed reductions (e.g. a 50% reduction for a PRNT evaluation point of PRNT_50_). Variability in plaque counts may result in the number of plaques counted under high dilutions exceeding the number formed when no sera is added. To avoid errors in the transformations, values of *p_d_* less than 0 were replaced with 0.001. As some laboratories use different PRNT evaluation points, we also calculated PRNTs over the range PRNT_40_ to PRNT_90_ [1,2].

As an alternative approach, some laboratories use non-linear regression approaches [3]. A popular method is the four-parameter model used by Prism 6 software (La Jolla, CA) for sigmoidal curves, which finds optimum values for the maximum and minimum plaque reductions, the slope of the linear part of the curve and the dilution of the inflexion point. To use this approach researchers need the plaque reductions from at least four dilutions.

***Bias and Mean Squared Error calculation***

For each experiment, we calculated PRNT using each of the models (probit, logit, cloglog regression and non-linear regression). Bias was suggested when there was a systematic difference between the PRNT estimates using the model and PRNT^sp^:

$$bias^{i}\left( x,v,p,m,y \right)=PRNT^{i}\left( x,v,p,m,y \right)-PRNT^{sp}(x,v,p,y)$$

where *PRNT^i^(x, v, p, m,y)* is the PRNT estimate from experiment *i* conducted with viral strain *v* (the five virus strains in Table 1) in serum pool *p* (either the high titer or the lower titer pool) estimated using model *m* (probit, logit, cloglog or four-parameter non-linear regression models) from assays performed in year *y* (2007, 2008, 2009, 2010, 2011, 2012 or 2013) at a PRNT evaluation point of x (varied from 40 to 90); *PRNT^sp^ (x,v,p,y)* is the estimate of the ‘true’titer, where *x* is the PRNT evaluation point, *v* is the viral strain, *p* is the serum pool and *y* is the year.

In addition, for each viral strain, for both the high and low titer pools, we calculated the mean squared error (MSE) of each parametric model using the following relationship:

$$MSE\left( x,v,p,m,y \right)=Variance\left( x,v,p,m,y \right)+Bias\left( x,v,p,m,y \right)^{2}$$

Where *Variance(x, v, p, m,y)* is the variance and *Bias(x, v, p, m,y)* is the mean bias in PRNT estimates using model *m* from all experiments conducted with viral strain *v* in serum pool *p* in year *y*. We reported an average MSE, bias ($\bar{B}\left( x,m \right)$) and variance ($\bar{V}\left( x,m \right)$) for each PRNT evaluation point and model, weighted by the number of experiments using each virus and serum pool.

The variance, bias and MSE of titers was calculated from individual sets of dilutions (i.e. no repeats) and from the single set of dilution repeats on the same plate. In addition, the variance between titers from the same plate was also calculated.

***Confidence interval calculation for bias and variance***

We used bootstrap resampling to calculate 95% confidence intervals for the average bias ($\bar{B}\left( x,m \right)$), variance ($\bar{V}\left( x,m \right)$) and MSE for each model and PRNT evaluation point. We recalculated the bias, variance and MSE over 1000 resamples, where individual sets of dilutions were the resampling unit (with replacement). 95% confidence intervals were calculated from the 2.5% and 97.5% quantiles of the resulting distributions.

***Precision in variance by number of repeat assays***

We calculated the width in the 95% confidence intervals of $\bar{V}\left( x,m \right)$ for the model m and PRNT evaluation point *x* that had the lowest mean-squared error for different number of repeat assays*.* Over 1,000 simulations, we randomly selected between 2 and 30 titer estimates from assays conducted in a single year on the same serum using the same viral strain and calculated the variance between the titer estimates. We then calculated 95% confidence intervals from the 2.5% and 97.5% quantiles from all simulations. The confidence interval width was the difference in these two values.

***Absolute titer confidence interval calculation***

Variability in titers between plates arises from plate-specific factors (that are identical between assays performed on the same plate) and non-plate-specific factors that are present irrespective of where the assay is performed.

*V_tot_ = V_ps_ + V_nps_*

where *V_tot_* is the total variance, *V_ps_* is the plate specific variance and *V_nps_* is the non-plate-specific variance (all on a logarithmic scale). *V_tot_* can be estimated from the variability in titers from single sets of dilutions performed on different plates, *V_nps_* can be estimated from the variance in titers from assays performed on the same plate. *V_ps_* is then the difference between the two. As assays performed on the same plate are perfectly correlated for plate-specific factors, increasing the number of repeats on the same plate can only reduce non-plate-specific variance. Therefore where repeats are performed on the same plate, the total variance becomes:

*V_tot_ = V_ps_ + V_nps_/n*

where *n* is the number of repeats performed on the same plate. Where repeats are performed on different plates, the total variance becomes:

*V_tot_ = V_ps_/n + V_nps_/n*

In each case, 95% asymptotic confidence intervals can be calculated.

Log-scale:

$$\log_{10} \left( T \right)+B\pm1.96\cdot\sqrt{V_{tot}}$$

where *T* is the titer estimate and *B* is the estimated bias using a particular model and PRNT evaluation point.

Linear-scale:

$${10}^{\log_{10} \left( T \right)+B\pm1.96\cdot\sqrt{V_{tot}}}$$

The confidence interval can be interpreted as the range of values that contain 95% of measured titers when the true titer is *T*.

***Relative titer confidence interval calculation***

Relative titers are calculated as the ratio of titers (on a linear scale) of two samples from the same individual. Where the samples are placed on different plates and a single set of dilutions are performed, the variance in the two titer estimates can be considered independent. The variance in the ratio of the titers therefore sums:

*V_rat_=V_s1_+V_s2_*

where *V_rat_* is the variance of the two samples, *V_s1_* is the variance of sample 1 and *V_s2_* is the variance of sample 2 (all on a logarithmic scale). As *V_s1_* and *V_s2_* will be approximately the same and each variance estimate is made up of plate-specific and non-plate-specific variance:

*V_rat_=2*(V_ps_ +V_nps_)*

However, when the samples are placed on the same plate, the variance in plate-specific factors are perfectly correlated. Therefore only variance from non-plate-specific factors (factors that are present irrespective of where the assays conducted) exists.

*V_rat_=2*V_nps_*

In each case we can calculate asymptotic 95% confidence intervals as follows:

Log-scale:

$$\log\left( T_{1} \right)-\log\left( T_{2} \right)\pm1.96*\surd V_{rat}$$

Linear scale:

$${10}^{\log\left( T_{1} \right)-\log\left( T_{2} \right)\pm1.96*\surd V_{rat}}$$

where T_1_ is the titer estimate from sample 1 and T_2_ is the titer estimate from sample 2. Note the non-centrality of the confidence interval on the linear scale (i.e., the ratio does not fall in the middle of the confidence interval).

***Multilevel model***

We constructed a multilevel model with a random intercept for each viral strain and serum pool combination (listed in Table 1):

$$y_{ij}=\gamma_{0}+\mu_{j}+\beta_{1}{Pass}_{i}+\beta_{2}{Cell}_{i}+\beta_{3}Age_{i}+\beta_{4}Density_{i}+\beta_{5}Year_{i}$$

where *y_ij_* is the log-transformed PRNT estimate for experiment *i* using serum pool *j*, *μ_j_* represents the random intercept for serum pool *j*, *Pass* is the total number of passages, *Cell* is a factor that represents either passages in C6/36 and LLC-MK2 cells, C6/36 and SM cells or C6/36 cells only. *Age* is the age of the virus stock at the time of the experiment (in years), *Density i*s the number of plaques in the reference well (where no sera is added) and *Year* is the year of the experiment. We assumed the errors in the model were normally distributed. The multi-level model was run using the statistical model (probit, logit, cloglog or four-parameter non-linear regression) and PRNT evaluation point with the lowest mean-squared error using titers estimated from a single set of dilution repeats from the same plate.

**References**

1. Russell PK, Nisalak A, Sukhavachana P, Vivona S (1967) A Plaque Reduction Test for Dengue Virus Neutralizing Antibodies. The Journal of Immunology 99: 285–290.

2. McArthur JH, Durbin AP, Marron JA, Wanionek KA, Thumar B, et al. (2008) Phase I clinical evaluation of rDEN4Delta30-200,201: a live attenuated dengue 4 vaccine candidate designed for decreased hepatotoxicity. The American Journal of Tropical Medicine and Hygiene 79: 678–684.

3. Puschnik A, Lau L, Cromwell EA, Balmaseda A, Zompi S, et al. (2013) Correlation between Dengue-Specific Neutralizing Antibodies and Serum Avidity in Primary and Secondary Dengue Virus 3 Natural Infections in Humans. PLoS Negl Trop Dis 7: e2274–e2274. doi:10.1371/journal.pntd.0002274.
